# Directed Evolution of Soluble α-1,2-Fucosyltransferase Using Kanamycin Resistance Protein as a Phenotypic Reporter for Efficient Production of 2'-Fucosyllactose

**DOI:** 10.4014/jmb.2209.09018

**Published:** 2022-10-17

**Authors:** Jonghyeok Shin, Seungjoo Kim, Wonbeom Park, Kyoung Chan Jin, Sun-Ki Kim, Dae-Hyuk Kweon

**Affiliations:** 1 Department of Integrative Biotechnology, College of Biotechnology and Bioengineering, Sungkyunkwan University, Seoburo 2066, Suwon, Gyeonggi 16419, Republic of Korea; 2 Carl R. Woese Institute for Genomic Biology, University of Illinois at Urbana-Champaign, Urbana, IL 61801, USA; 3 Department of Food Science and Technology, Chung-Ang University, Anseong, Gyeonggi 17546, Republic of Korea

**Keywords:** 2′-fucosyllactose, α-1,2-fucosyltransferase, directed evolution, solubility biosensor, kanamycin resistance, *Escherichia coli*

## Abstract

2'-Fucosyllactose (2'-FL), the most abundant fucosylated oligosaccharide in human milk, has multiple beneficial effects on human health. However, its biosynthesis by metabolically engineered *Escherichia coli* is often hampered owing to the insolubility and instability of α-1,2-fucosyltransferase (the rate-limiting enzyme). In this study, we aimed to enhance 2'-FL production by increasing the expression of soluble α-1,2-fucosyltransferase from *Helicobacter pylori* (FucT2). Because structural information regarding FucT2 has not been unveiled, we decided to improve the expression of soluble FucT2 in *E. coli* via directed evolution using a protein solubility biosensor that links protein solubility to antimicrobial resistance. For such a system to be viable, the activity of kanamycin resistance protein (Kan^R^) should be dependent on FucT2 solubility. Kan^R^ was fused to the C-terminus of mutant libraries of FucT2, which were generated using a combination of error-prone PCR and DNA shuffling. Notably, one round of the directed evolution process, which consisted of mutant library generation and selection based on kanamycin resistance, resulted in a significant increase in the expression level of soluble FucT2. As a result, a batch fermentation with the ΔL M15 pBCGW strain, expressing the FucT2 mutant (F#1–5) isolated from the first round of the directed evolution process, resulted in the production of 0.31 g/l 2'-FL with a yield of 0.22 g 2'-FL/g lactose, showing 1.72- and 1.51-fold increase in the titer and yield, respectively, compared to those of the control strain. The simple and powerful method developed in this study could be applied to enhance the solubility of other unstable enzymes.

## Introduction

Human milk oligosaccharides (HMOs) have multiple beneficial functions, such as the regulation of the human immune system and protection against pathogens, including viruses, bacteria, and protozoa [1]. Among the various oligosaccharides present in HMOs, 2'-fucosyllactose (Fuc-α1,2-Gal-β1,4-Glc; 2'-FL) is highly abundant and has attracted great interest as a functional food ingredient because of its pharmaceutical and nutraceutical properties [2, 3]. However, the milk of some women (non-secretor) does not contain 2'-FL owing to genetic variation [4].

A biosynthetic approach based on in vitro enzymatic reactions or metabolically engineered microorganisms is commonly considered an efficient and sustainable method for producing 2'-FL on a large scale [5]. Since in vitro multienzyme catalysis requires purified reagents and enzymes that increase production costs, production of 2'-FL using engineered microbes has been proven to be cost effective [5, 6]. In engineered *Escherichia coli* [7, 8] and *Saccharomyces cerevisiae* [9, 10] capable of producing 2'-FL, lactose is fucosylated by a heterologous α-1,2-fucosyltransferase using guanosine 5'‐diphosphate (GDP)-L-fucose as a fucose donor. However, despite several attempts to construct high-performance strains, the 2'-FL titers are often reported to be low because of the insoluble and unstable nature of α-1,2-fucosyltransferase under process conditions [11, 12].

Fusion protein technologies [13], chaperone co-expression [14], directed evolution [15], promoter optimization [16], and machine learning [17] have been used to improve solubility of target proteins. We previously developed an inducible plasmid display system to improve the solubility of α-1,2-fucosyltransferase from *Helicobacter pylori* (FucT2) [18]. In this system, the C-terminal end of FucT2 and the N-terminal end of Oct-1 DNA-binding domain were fused to the N-terminal and C-terminal domains of an intein, respectively. Inteins are protein segments capable of mediating protein *trans*-splicing, which refers to the ligation of flanking extein into a new protein [19]. The efficiency of protein *trans*-splicing and FucT2 display on a plasmid were designed to be dependent on FucT2 solubility. However, soluble FucT2 mutants with improved activity were not obtained, mainly because the efficiency of FucT2 display on a plasmid was primarily dependent on induction time and expression levels of protein *trans*-splicing domains [18, 20].

**Fig. 1:**
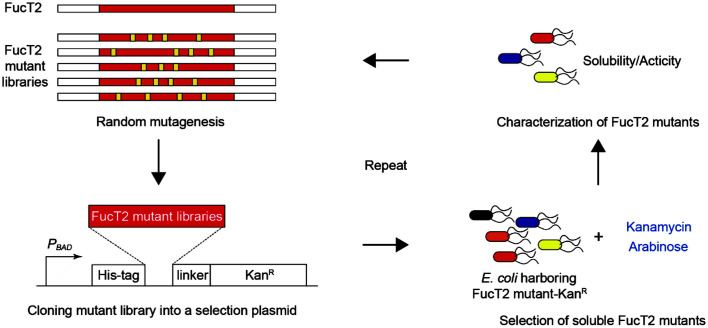
A schematic diagram representing overall process of directed evolution. FucT2 mutant libraries were generated via a combination of error-prone PCR and DNA shuffling. The expression of the fusion protein (FucT2-Kan^R^) consisting of FucT2 and kanamycin (Kan) resistance protein (Kan^R^) was under the transcriptional control of the *P_BAD_* promoter. The amino acid sequence of the linker was KSAAGT. Kan-resistant *E. coli* Top10 cells expressing FucT2-Kan^R^ were isolated from solid LB medium containing 2 g/l L-arabinose and 10–200 μg/ml Kan. Finally, the solubility and activity of FucT2 mutants were characterized, or they were used as substrates for the next round of evolution.

To overcome this limitation, we used a biosensor that links protein solubility to antimicrobial resistance. For such a system to be viable, the antibiotic resistance of cells containing the biosensor should be directly correlated with FucT2 solubility. To this end, aminoglycoside-3'-phosphotransferase (*i.e.*, kanamycin resistance protein) was fused to the C-terminus of the mutant libraries of FucT2 (Fig. 1). In this system, the biosensor is rendered non-functional when FucT2 mutants are expressed in an insoluble form, rendering the cells containing the corresponding constructs susceptible to kanamycin. In contrast, soluble FucT2 mutants confer high levels of kanamycin resistance to cells.

In this study, a protein solubility-based selection strategy for directed evolution was developed to allow for a high-throughput analysis of complex libraries within a short time and to enhance 2'-FL production in recombinant *E. coli*.

## Materials and Methods

### Strains and Plasmids

All the strains and plasmids used in this study are listed in Table 1. *E. coli* TOP10 was used for genetic manipulation and directed evolution of FucT2, and *E. coli* ΔL M15 [11] was used for 2'-FL production. A ligase-independent molecular cloning method was used [21]. To construct pSel2-FucT2, pSel2-FucT2 I and pSel2-FucT2 V primers were used to amplify the coding sequence of FucT2 and linearize the plasmid pSel2 (Addgene, USA), respectively. After the DNA fragments of the vector and the insert were amplified using Pfu polymerase (Elpis-Biotech, Daejeon, Korea), they were ligated using T4 DNA polymerase (New England Biolabs, USA).

pColAduet-FucT2 and pColAduet-FucT2mut plasmids were designed to contain the genes encoding wild-type (WT) FucT2 and its mutants, respectively, for production of 2'-FL in *E. coli*. To construct pColAduet-FucT2mut, pColAduet-FucT2 I and pColAduet-FucT2 V primers were used to amplify the coding sequence of FucT2 and to linearize the plasmid pColAduet-1 (Novagen, Germany), respectively. The two DNA fragments were amplified and ligated as described above. The primers used for plasmid construction are listed in Table S1.

### Kanamycin Sensitivity Assay

*E. coli* TOP10 harboring pSel2 or pSel2-FucT2 was precultured overnight at 37°C and 200 ×*g* in Luria–Bertani (LB) medium (10 g/l tryptone, 5 g/l yeast extract, and 10 g/l sodium chloride). The precultured cells were streaked on solid selection medium containing 17 g/l Miller Hilton agar (Sigma, USA), 0.4 g/l L-arabinose, 50 μg/ml ampicillin, and 0–200 μg/ml kanamycin and were incubated overnight at 37°C.

### Construction of FucT2 Mutant Library

To construct a primary FucT2 mutant library, the gene coding for FucT2 was amplified with pSel2-FucT2 I FW and BW primers using a GeneMorph II Random Mutagenesis Kit (Agilent Technologies, USA) according to the manufacturer’s instructions. The resulting PCR products were ligated with the pSel2 plasmid to construct an intermediate plasmid, and additional mutations were introduced using a JBS DNA-Shuffling Kit (Jena Bioscience, Germany), as specified by the manufacturer. Briefly, the primary FucT2 mutant library present in the intermediate plasmid was amplified using Pfu polymerase (Elpis Biotech, Korea). The products were purified using a gel-prep kit (Elpis-Biotech) and digested with DNase I. The digested DNA fragments were reassembled using Taq polymerase (Elpis-Biotech) without primers, and reassembled *FucT2* was amplified using pSel2-FucT2 I FW and BW primers. The amplified FucT2 mutant library was ligated with the pSel2 plasmid to generate pSel2-FucT2mut.

### Selection of FucT2 Mutants with Improved Solubility

10^5^–10^6^ *E. coli* TOP10 cells harboring FucT2 mutant library were plated on solid selection medium containing 17 g/l Miller Hilton agar, 0.4 g/l L-arabinose, 50 μg/ml ampicillin, and 0–200 μg/ml kanamycin and were incubated overnight at 37°C. Kanamycin was added at final concentrations of 10, 25, 50, and 100 μg/ml for the first, second, third, and fourth rounds, respectively. Fifteen large colonies were selected in each round of directed evolution.

### Preparation of Proteins

*E. coli* Top10 strains harboring pSel2-FucT2 or pSel2-FucT2mut were precultured overnight at 37°C and 200 rpm in LB medium. After harvesting the cells, cell pellets were used for inoculation. A batch was cultured in a 250-ml baffled flask containing 50 ml of LB medium and 50 μg/ml ampicillin at 37°C and 200 ×*g*. The cells were cultivated for 3 h in the absence of an inducer, and then cultivated for an additional 3 h after the addition of 2 g/l L-arabinose. After harvesting the cells, the cell pellets were resuspended in phosphate-buffered saline (pH 7.4) and disrupted using an ultrasonic machine (Sonics & Materials Inc., USA) on ice. The total, soluble, and insoluble fractions of intracellular proteins were prepared as previously described [12].

### Analysis of Protein Expression

Protein samples were separated using 12% (w/v) sodium dodecyl sulfate-polyacrylamide gel electrophoresis and then transferred to polyvinylidene difluoride membranes. In immunoblot assays, His_6_-fusion proteins were detected using an anti-polyhistidine antibody (Sigma-Aldrich), as specified by the manufacturer. Band intensities were quantified using densitometry software (GelAnalyzer 19.1, Hungary).

### Culture Conditions and Analytical Methods

*E. coli* ΔL M15 strain [11] harboring pBCGW [22] was used as a host for 2'-FL production. Batch fermentation was performed in a 250-ml baffled flask containing 50 ml of a defined medium [13.5 g/l KH_2_PO_4_, 4 g/l (NH_4_)_2_HPO_4_, 1.7 g/l citric acid, 1.4 g/l MgSO_4_·7H_2_O, and 10 ml/l trace element solution (10 g/l FeSO_4_·7H_2_O, 2.25 g/l ZnSO_4_·7H_2_O, 1.0 g/l CuSO_4_·5H_2_O, 0.35 g/l MnSO_4_·H_2_O, 0.23 g/l Na_2_B_4_O_7_·10H_2_O, 0.1 g/l (NH_4_)_6_Mo_7_O_24_·4H_2_O, and 2.0 g/l CaCl_2_·2H_2_O), pH 6.8] with 20 g/l glycerol and 50 μg/ml ampicillin at 37°C and 200 rpm. At the mid-exponential growth phase, isopropyl β-D-1-thiogalactopyranoside (IPTG) and lactose were added at final concentrations of 0.1 mM and 5 g/l, respectively. After IPTG induction, the temperature was increased to 25°C.

Lactose, glycerol, and 2'-FL concentrations were determined using high-performance liquid chromatography (Waters Corporation, USA). Metabolites were separated using a Rezex ROA-Organic Acid H^+^ column (Phenomenex, USA) in isocratic mode at a constant temperature (50°C) and a flow rate of 0.6 ml/min in 0.01 N H2SO_4_, and then passed through a refractive index detector (Fig. S1).

### Statistical Analysis

Statistical methods were not used to determine sample size. Randomization and blind tests were not performed to evaluate the experimental procedures and results. Each experiment was performed in triplicate. Numerical analyses were performed using GraphPad Prism 5 (GraphPad Software Inc., San Diego, CA, USA). The Student’s *t*-test was used to determine numerical significance. The results were considered significant when *p* < 0.05.

## Results

### Expression of an Insoluble FucT2-Kan^R^ Fusion Protein Comprising FucT2 and Kanamycin Resistance Protein

**Fig. 2:**
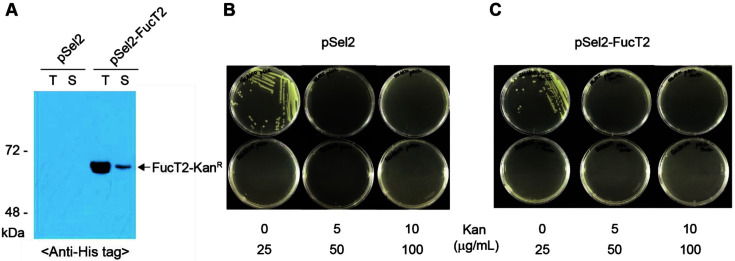
The loss of Kan resistance owing to FucT2 insolubility. (**A**) Western blot analysis was used to confirm the expression of FucT2-Kan^R^. Cell lysates were prepared from recombinant *E. coli* Top10 containing pSel2 and pSel2-FucT2 and fractionated into total (T) and soluble (S) fractions. (**B, C**) Recombinant *E. coli* Top10 containing pSel2 (**B**) and pSel2-FucT2 (**C**) were streaked on selection medium containing various concentrations of Kan.

To construct an expression plasmid (pSel2-FucT2) for the fusion protein (FucT2-Kan^R^) consisting of FucT2 and kanamycin resistance protein (Kan^R^), the coding sequence of FucT2 was cloned into the pSel2 plasmid containing the *P_BAD_* promoter, in which protein expression was induced by adding L-arabinose. The recombinant *E. coli* Top10 harboring pSel2 and pSel2-FucT2 was grown at 37°C for 3 h in the presence of 2 g/l L-arabinose, and the total and soluble protein fractions were analyzed by Western blotting. Most FucT2-Kan^R^ were not expressed as soluble forms (Fig. 2A). Densitometric analysis showed only 10% FucT2-Kan^R^ expression in the soluble form.

To determine the minimum concentration of kanamycin that completely inhibits the growth of the *E. coli* strain expressing FucT2-Kan^R^, *E. coli* Top10 harboring pSel2 and pSel2-FucT2 was grown on solid medium containing various concentrations of kanamycin. In the DNA sequences of the pSel2 plasmid, frameshift mutations were introduced in the coding regions of Kan^R^. Consequently, *E. coli* Top10 harboring pSel2 was not able to grow in medium containing 5–100 μg/ml kanamycin (Fig. 2B). Insertion of the coding sequence of FucT2 into the pSel2 plasmid resulted in the formation of FucT2-Kan^R^ that was designed to counter the effects of frameshift mutations (Fig. S2). Accordingly, the FucT2-Kan^R^ fusion protein would catalyze the conversion of kanamycin into an inactive form if it were expressed in a soluble form. However, the growth of *E. coli* Top10 expressing FucT2-Kan^R^ was not detected in the kanamycin-containing medium to be tested (Fig. 2C). This result indicates that the expression of soluble FucT2-Kan^R^ was too low to confer resistance to kanamycin, even at a low kanamycin concentration (5 μg/ml). Therefore, 10 μg/ml was selected as the basal concentration for further experiments on the directed evolution of FucT2.

### Construction of a FucT2 Mutant Library and Selection of FucT2 Mutants

In this study, a combination of error-prone PCR and DNA shuffling was used to obtain FucT2 variants with improved solubility. A primary FucT2 mutant library constructed via error-prone PCR (Fig. S3B) was re-amplified using Pfu polymerase (lane 2, Fig. S3C). After digesting the resulting PCR products with DNase I (lane 3, Fig. S3C), DNA fragments were reassembled into a full-length gene in the absence of primers (lane 4, Fig. S3C). The 903-bp DNA fragments containing FucT2 mutant libraries were amplified with pSel2-FucT2 I FW and BW primers and then ligated into the pSel2 plasmid to construct pSel2-FucT2mut (lane 5, Fig. S3C).

Fifteen large colonies of *E. coli* Top10 harboring pSel2-FucT2mut and grown on solid medium containing 10 μg/ml kanamycin were selected, and DNA sequences of the gene coding for FucT2 mutants were analyzed. Among various FucT2 mutants, we decided to focus on full-length variants, excluding mutants with frameshift, truncation, and/or overlapping. Based on this criterion, two FucT2 mutants were selected from the first round of the overall process represented in Fig. 1 and mixed at the same ratio for use as the template for the next round of directed evolution and selection. Finally, 15 FucT2 mutants were collected after four rounds of the overall process (Table 2).

### Soluble Expression of FucT2 via Kan^R^-Mediated Directed Evolution

**Fig. 3:**
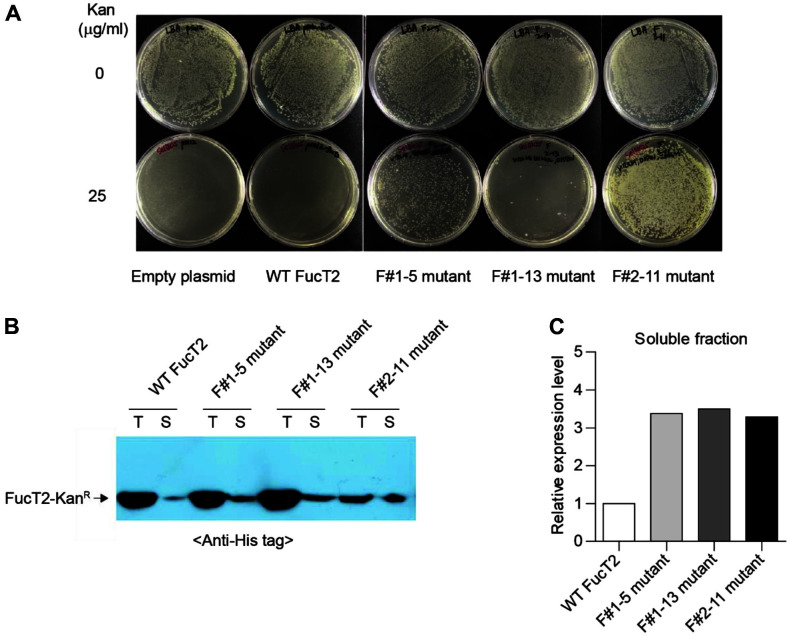
Validation of Kan^R^-mediated directed evolution for improving the expression of soluble FucT2. (**A**) Recombinant *E. coli* Top10 expressing wild-type (WT) FucT2 and its mutants (F#1-5, F#1-13, and F#2-11) were grown on selection medium with and without 25 μg/ml Kan. (**B**) Western blot analysis was used to confirm FucT2-Kan^R^ expression. Cell lysates were prepared from recombinant *E. coli* Top10 expressing WT FucT2 and its mutants, and divided into total (T) and soluble (S) fractions. (**C**) Comparison of relative expression of soluble FucT2-Kan^R^ detected in Fig. 3B.

F#1-5, F#1-13, and F#2-11 mutants were selected from the first and second rounds of directed evolution. Before measuring the expression of WT FucT2 and its mutants selected via directed evolution using Kan^R^ as a biosensor motif, colony-forming units (CFUs) of *E. coli* Top10 strains expressing WT FucT2 and its three mutants (F#1-5, F#1-13, and F#2-11) were compared on LB medium with and without 25 μg/ml kanamycin. The CFUs of all strains were almost identical in LB medium without kanamycin (Fig. 3A). While colony formation by *E. coli* strains harboring the empty plasmid (pSel2) and pSel2-FucT2 was not observed on LB medium containing kanamycin, *E. coli* strains expressing F#1-5, F#1-13, and F#2-11 mutants were able to grow in the presence of 25 μg/ml kanamycin (Fig. 3A). Notably, CFUs of *E. coli* strain expressing the F#2-11 mutant, which were isolated from the second round of directed evolution, were much higher than those of *E. coli* strains expressing F#1-5 and F#1-13 mutants. This result indicated that the kanamycin resistance potential of FucT2-Kan^R^ containing F#1-5 or F#1-13 mutants was further enhanced via the second round of directed evolution.

Next, we evaluated whether the expression of soluble FucT2 could be enhanced via Kan^R^-mediated directed evolution. Although total expression of the F#2-11 mutant was slightly lower than that of the WT FucT2, and F#1-5 and F#1-13 mutants, the expression of soluble FucT2-Kan^R^ by the F#1-5, F#1-13, and F#2-11 mutants was significantly higher than that by the WT (Fig. 3B). The decrease in total expression level of the F#2-11 mutant is likely due to the dynamic transtriptional response of *E. coli* to the soluble property of the F#2-11 mutant. This result is consistent with a previous study which reported that expression levels of genes involved in protein synthetic pathway were altered due to the change of expression level of inclusion bodies [23]. Analysis of the band intensities by Western blotting showed that the level of soluble FucT2-Kan^R^ produced in the presence of F#1-5, F#1-13, and F#2-11 was approximately 3-fold greater than that by the WT (Fig. 3C). Therefore, evolutionary engineering of FucT2 using Kan^R^ as a phenotypical reporter successfully enhanced the expression of soluble FucT2.

### Enhanced Production of 2'-FL by Engineered *E. coli* Expressing FucT2 Mutants with Improved Solubility

**Fig. 4:**
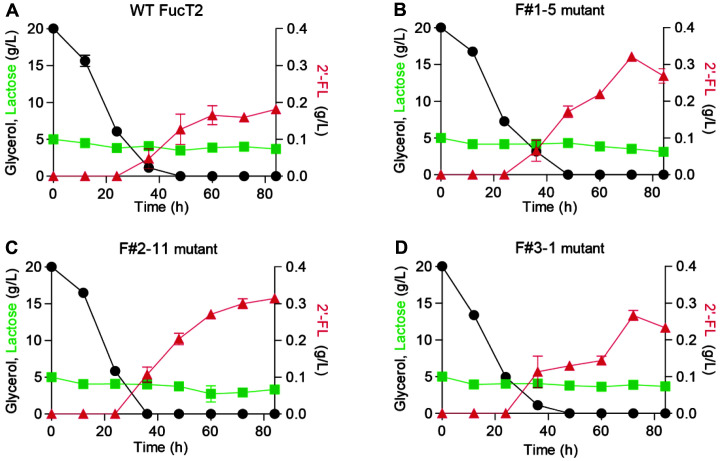
Comparison of 2'-FL production in engineered *E. coli* strains expressing wild-type (WT) FucT2 (**A**) and F#1-5 (**B**), F#2-11 (**C**), and F#3-1 (**D**) mutants. At the mid-exponential growth phase, IPTG and lactose were added at final concentrations of 0.1 mM and 5 g/l, respectively. Results are the mean of duplicate experiments and error bars indicate standard deviation.

An engineered *E. coli* strain BL21star (DE3) ΔL M15 harboring pBCGW (ΔL M15 pBCGW) was previously constructed for de novo production of 2'-FL [11]. Specifically, genes coding for enzymes involved in producing GDP-L-fucose (a precursor of 2'-FL) were overexpressed along with attenuation of the lac operon to redirect carbon flux from lactose to 2'-FL. A previous study reported that the ΔL M15 pBCGW strain expressing WT FucT2 produced 0.16 g/l 2'-FL with a yield of 0.091 g 2'-FL/g lactose [11]. Therefore, we decided to evaluate whether expression of the four FucT2 mutants (F#3-1, F#3-9, F#4-6, and F#4-19) from the third and fourth rounds of Kan^R^-mediated directed evolution, in addition to those from the first and second rounds (F#1-5, F#1-13, and F# 2-11) in the ΔL M15 pBCGW strain, could enhance 2'-FL production. Before comparing 2'-FL production, we analyzed total and soluble expression of the WT FucT2 and its mutant in the absence of Kan^R^ fusion. The expression level of soluble FucT2 by the mutants was higher than that by the WT FucT2 in the absence of Kan^R^ (Fig. S4). Notably, we observed efficient 2'-FL production by *E. coli* strains expressing F#1-5, F#2-11, and F#3-1 mutants (Fig. 4). The cell growth patterns of all *E. coli* strains were almost identical (data not shown). The ΔL M15 pBCGW strains expressing F#1-5, F#2-11, and F#3-1 mutants were able to produce 0.27–0.31 g/l 2'-FL with a yield of 0.17–0.22 g 2'-FL/g lactose, showing 1.46–1.72- and 1.12–1.51-fold increases in the titer and yield, respectively, when compared to those of the control strain expressing WT FucT2. Collectively, the expression of FucT2 mutants evolved using Kan^R^ as the phenotypical reporter led to a significant increase in the production titer and yield of 2'-FL in *E. coli*.

## Discussion

α-1,2-Fucosyltransferase is the rate-limiting enzyme in *E. coli*-based production of 2'-FL [11]. Therefore, in this study, we aimed to enhance its solubility and functional expression of 2'-FL. The efficient construction of desired mutants via rational design is not possible without prior knowledge of the structure and mechanism of action of the target enzymes [24]. Given the absence of structural information on FucT2, directed evolution was used to improve the expression of soluble FucT2.

The overall process of directed evolution consists of generating a mutant library and high-throughput screening and/or selection (HTSS). Although mutant libraries with controllable numbers of mutations can be generated via modern mutagenesis techniques, such as error-prone PCR and DNA shuffling, the real bottleneck for solubility engineering lies in establishing HTSS for mutant isolation [25]. Using a biosensor of protein solubility may overcome such bottlenecks, thereby increasing the probability of selecting desired mutants. A typical biosensor of protein solubility consists of target and phenotypical reporter proteins [26]. Since these two are expressed as a fusion protein, the activity of the reporter is affected by the solubility of the target protein. Chloramphenicol acetyltransferase (CAT), which confers resistance to chloramphenicol, is a frequently used reporter protein owing to its easily detectable enzymatic activity [27]. Cells expressing insoluble and/or unstable proteins fused to CAT were unable to grow in the presence of chloramphenicol, whereas mutants with improved solubility and/or stability could be isolated from solid medium containing chloramphenicol. Examples of successful applications of CAT-based biosensors include isolation of mitochondrial Acytochrome P450 [28], imidazole glycerol phosphate synthase [29], and interleukin-15 [30] mutants with improved solubility and/or stability. In addition to CAT, green fluorescent protein (GFP) has been used as a phenotypical reporter protein. In recent studies, the solubility and/or stability of L-serine/L-threonine exchanger [31] and esterase [32] were significantly improved via the GFP-mediated directed evolution coupled with fluorescence size-exclusion chromatography analysis and fluorescence-activated cell sorting, respectively. In this system, variants with high levels of soluble protein expression can confer GFP with strong fluorescence, whereas low stability and/or solubility of target proteins results in improper folding of GFP which in turn lead to a decrease in the intensity of fluorescence.

For the biosensor with CAT to be viable, the activity of CAT should be solely dependent on FucT2 solubility. However, CAT activity in the fusion protein is dependent on its quaternary structure in addition to the solubility of target proteins because the homotrimeric CAT is catalytically active only as a trimer [33]. Therefore, Kan^R^ was employed in this study as an alternative phenotypic reporter. Although Kan^R^ thermodynamically favors dimers of identical subunits, dimerization does not affect its activity [34].

We noted that a single round of the overall directed evolution process enabled the expression of soluble FucT2, which resulted in a significant increase in 2'-FL production. However, the increase in FucT2 activity was not proportional to the extent of FucT2 solubility. Although the mechanism remains to be investigated, we hypothesize that one of the reasons for this might be due to the activity-solubility trade-offs, or rather, the negative correlation between activity and solubility [35, 36]. Since amino acids in enzymes have multiple roles, the adjustment of one physical parameter could influence other properties [37, 38]. Among seven FucT2 mutants with improved solubility, only three (F#1-5, F#1-13, and F# 2-11) exhibited higher activity than that of the WT FucT when expressed in ΔL M15 pBCGW strain (Fig. S5). In addition, the activity of Kan^R^ was not directly proportional to the expression level of soluble FucT2-Kan^R^ by the mutants. These results are consistent with those of previous studies that reported a decrease in enzyme activity of the mutants with improved solubility and/or stability [37-39]. In our ongoing research, we are striving to identify the key mutations that play crucial roles in solubility enhancement and/or mutations that enhance solubility without negatively affecting activity. The FucT2 structure predicted using AlphaFold [40], an artificial intelligence method for predicting protein structures, showed that the key mutation sites (V33, G84, D120, and I287) were present on the surface of FucT2 (Fig. S6). A previous study reported that hydrophobic amino acids on the surface of recombinant proteins played an important role in the formation of inclusion body [41]. Therefore, we speculated that the V33M, G84R, D120N, and I287M mutations enhanced expression level of soluble FucT2 by decreasing formation of inclusion body.

In conclusion, in this study, we aimed to enhance the expression of soluble FucT2 via directed evolution for the efficient production of 2'-FL. To this end, Kan^R^ was employed in this study as a phenotypic reporter, whose activity is affected by the solubility of FucT2. Three FucT2 mutants (F#1-5, #F2-11, and F#3-1) with improved solubility and activity were successfully isolated. Finally, ΔL M15 pBCGW strain expressing F#1-5, #F2-11, and F#3-1 produced 0.27–0.31 g/l 2'-FL, showing a 1.46–1.72 fold increase in 2'-FL titer, compared to those of the control strain. Therefore, our approach could be applied to other biotransformation processes that include highly unstable and insoluble enzymes.

## Supplemental Materials

Supplementary data for this paper are available on-line only at http://jmb.or.kr.

## Figures and Tables

**Table 1:** *Escherichia coli* strains and plasmids used in this study.

Name	Description	Reference
*E. coli*		
TOP10	F^–^ *mcrA* Δ(*mrr-hsdRMS-mcrBC*) Φ80*lacZ*ΔM15 Δ*lacX74 recA1 ara*D139 Δ(*ara-leu*)*7697 galU galK rpsL* (Str^R^) *endA1 nupG*	Invitrogen (USA)
ΔL M15	BL21 star (DE3) Δ*lacZYA Tn7::lacZΔM15*	[11]
Plasmids		
pSel2	*P_BAD_* promoter-Kan^R^ expression system, ColEI origin, Amp^R^	[42]
pSel2-FucT2	pSel2 + wild type FucT2	This study
pSel2-FucT2mut	pSel2 + FucT2 mutants	This study
pColAduet-1	Two *T7* promoters, ColA origin, Kan^R^	Novagen (Germany)
pColAduet-FucT2	pColAduet + wild type FucT2 from *H. pylori*	AP Technology (Republic of Korea)
pColAduet-FucT2mut	pColAduet + FucT2 mutants	This study

**Table 2:** A list of FucT2 mutants isolated from five rounds of directed evolution process.

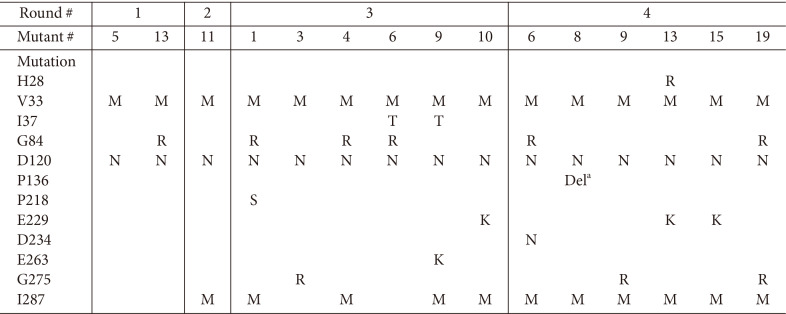

^a^Del: amino acid deletion
