# Supplementary material for: Directed Evolution of Soluble α-1,2-Fucosyltransferase Using Kanamycin Resistance Protein as a Phenotypic Reporter for Efficient Production of 2'-Fucosyllactose
Source: J Microbiol Biotechnol. 2022 Oct 17;32(11):1471–8. doi: 10.4014/jmb.2209.09018 (PMC9720067; doi:10.4014/jmb.2209.09018)
Supplement: Supplementary file 1 [file jmb-32-11-1471-supple.pdf]

J. Microbiol. Biotechnol.
https://doi.org/10.4014/jmb.2209.09018

Supplementary Materials

Table S1. A list of primers used in this study.

Name	Sequence (5′ → 3′)
pSel2-FucT2 I FW	GGATCCGAGCTCGAGATGGCTTTTAAGGT
pSel2-FucT2 I BW	GTACCAGCTGCAGATTTAGCGTTATACTTTTGGGA
pSel2-FucT2 V FW	ATCTGCAGCTGGTACCCGGCCGA
pSel2-FucT2 V BW	CTCGAGCTCGGATCCCCATCGATC
pColAduet-FucT2 I FW	AGGAGATATACCATGGCTTTTAAGGTGGTGCAAATTTGCGGAGGGCTT
pColAduet-FucT2 I BW	AGGCGCGCCGAGCTCTTAAGCGTTATACTTTTGGGATTTTACCTCAAAATGGGATTCTATTT TCA
pColAduet-FucT2 V FW	GAGCTCGGCGCGCCTGCAG
pColAduet-FucT2 V BW	CATGGTATATCTCCTTATTAAAGTTAAACAAAATTATTTCTACAGGGGAATTGTTA

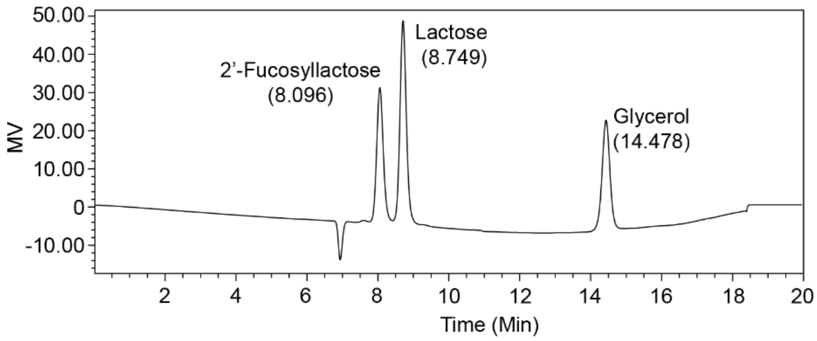

Supplementary figure 1. Representative HPLC chromatogram of standard compounds (2'-FL, lactose, and glycerol). A standard solution containing 2.5 g/L 2'-FL, lactose, and glycerol was analyzed by high-performance liquid chromatography equipped with a Rezex ROA-Organic Acid H^+ column.

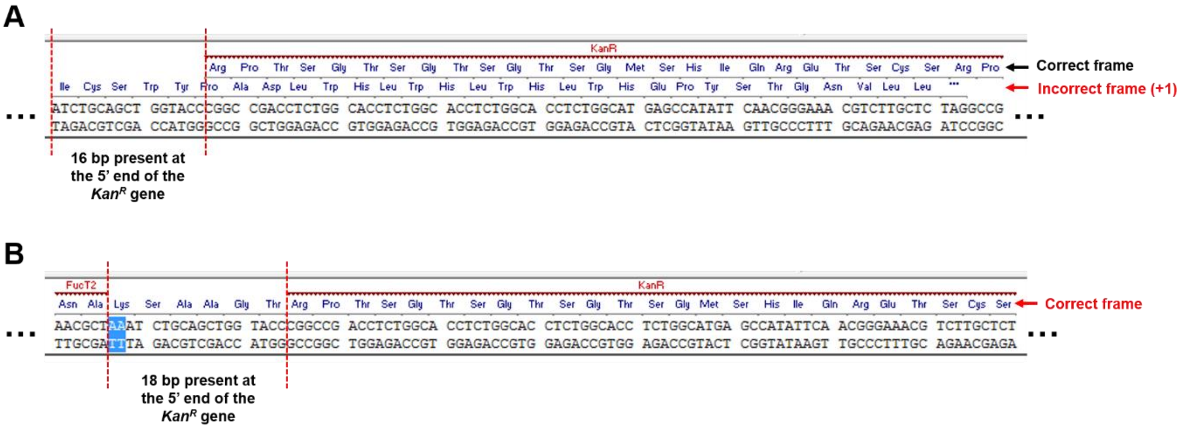

Supplementary figure 2. DNA sequences in pSel2 (A) and pSel2-FucT2 (B) plasmids. (A) The frameshift mutations in the coding regions of Kan^R resulted in formation of a truncated form of Kan^R with incorrect frame. (B) Insertion of the coding sequence of FucT2 as well as additional “AA” sequences (highlighted in blue) into the pSel2 plasmid resulted in the formation of FucT2-Kan^R that counter the effects of frameshift mutations.

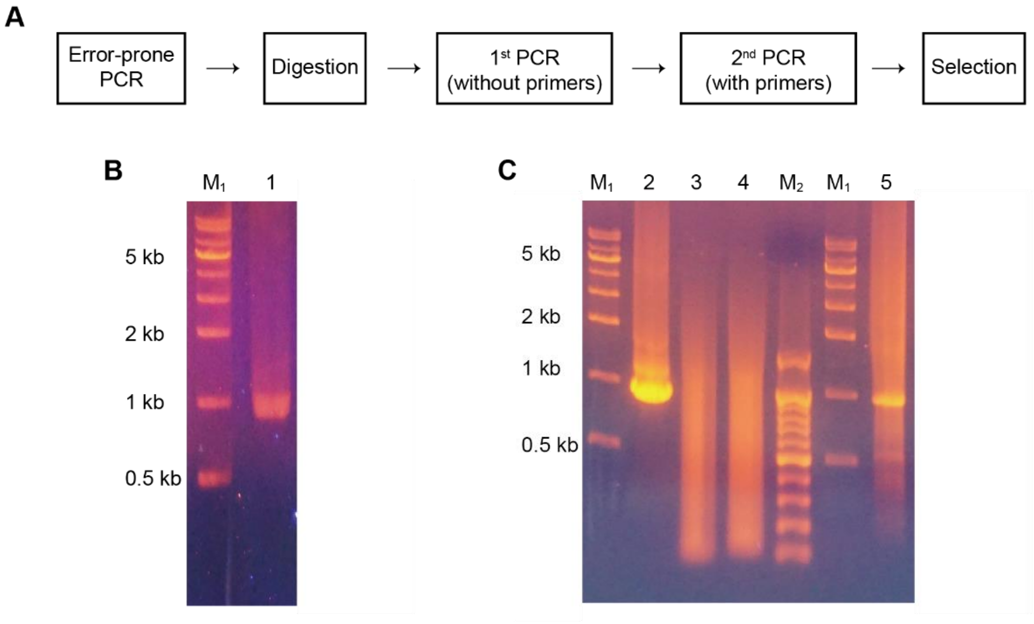

Supplementary figure 3. Agarose gel electrophoresis to confirm the construction of FucT2 mutant libraries. The mutant libraries were generated via a combination of error-prone PCR (A) and DNA shuffling (B). M_1, 1 kb DNA marker; M_2, 100 bp DNA marker, 1, the product of error-prone PCR; 2, the reamplified *FucT2* gene using pfu polymerase; 3, the DNA fragments digested by DNase I treatment; 4, the product of 1st PCR in the absence of primers; 5, the product of 2nd PCR in the presence of primers.

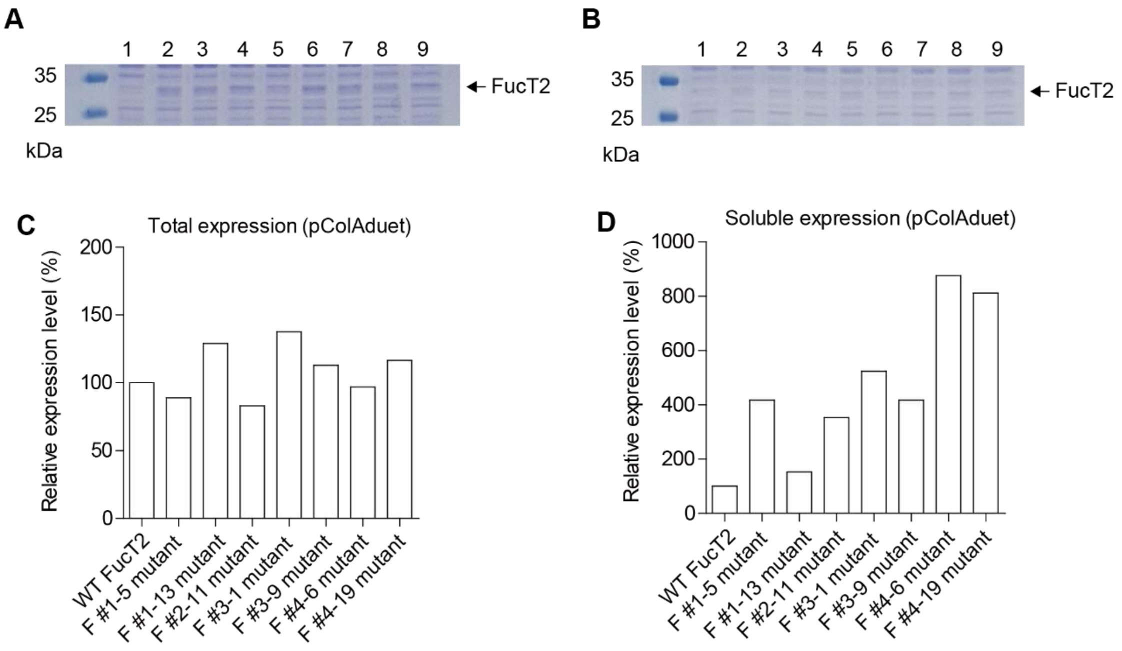

Supplementary figure 4. SDS-PAGE analysis to confirm FucT2 expression. Cell lysates were prepared from recombinant *E. coli* Top 10 expressing WT FucT2 and its mutants, and divided into total (A and C) and soluble (B and D) fractions. (A and B) SDS-PAGE analysis. 1, Empty plasmid; 2, WT FucT2; 3, F#1-5 mutant; 4, F#1-13 mutant; 5, F#2-11 mutant; 6, F#3-1 mutant; 7, F#3-9 mutant; 8, F#4-6 mutant; and 9, F#4-19 mutant. (C and D) Comparison of relative expression of FucT2 detected in Fig. S4A and B.

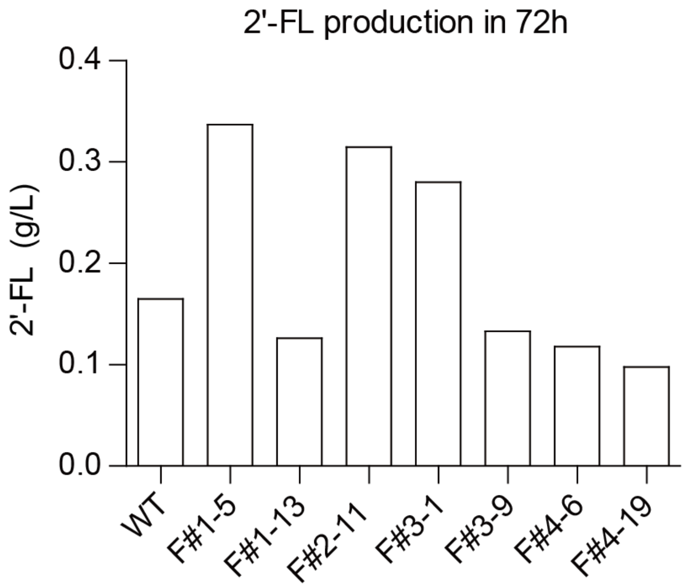

Supplementary figure 5. Comparisons of 2′-FL production in engineered *E. coli* strains expressing the wild type (WT) FucT2 and its mutants (F#1-5, F#1-13, F#2-11, F#3-1, F#3-9, F#4-6, and F#4-19). At the mid-exponential growth phase, IPTG and lactose were added at final concentrations of 0.1mM and 5 g/L, respectively.

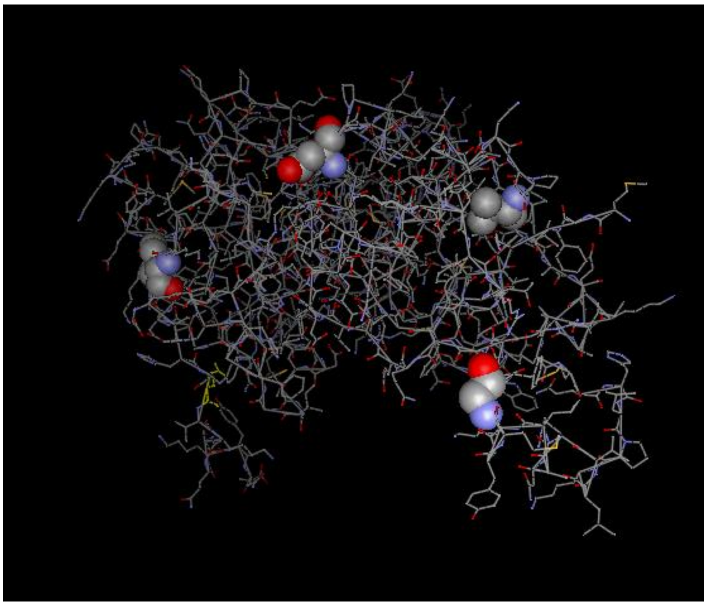

Supplementary figure 6. The structure of FucT2 predicted using AlphaFold.

The V33, G84, D120, and I287 residues were represented using spaced-filling model.
